# Supplementary material for: A multi-grained symmetric differential equation model for learning protein-ligand binding dynamics
Source: Nat Commun. 2025 Dec 30;17:1049. doi: 10.1038/s41467-025-67808-z (PMC12848303; doi:10.1038/s41467-025-67808-z)
Supplement: Supplementary file 1 — Supplementary Information [file 41467_2025_67808_MOESM1_ESM.pdf]

Supplementary Information

A Preliminaries and Related Works

A.1 Preliminaries on Molecular Dynamics

Molecular dynamics (MD) simulations predict how every atom in a molecular system moves over time, which is determined by the interatomic interactions following Newton's second law. Such a molecular system includes small molecules [1, 2], proteins [3, 4, 5], polymers [6], crystals [7], and protein-ligand complexes [8, 9]. Typically, an MD simulation is composed of two main steps, *i.e.*, (1) the energy and force calculation and (2) integration of the equations of motion governed by Newton's second law of motion, using the initial conditions and forces calculated in step (1). As the initial condition, the initial positions and velocities are given for all the particles (*e.g.*, atoms) in the molecular system; the MD simulation repeats the two steps to get a trajectory. Such MD simulations can be used to calculate the equilibrium and transport properties of molecules, materials, and biomolecular systems [10].

In such an MD simulation, one key factor is estimating the forces on each atom. The function that gives the energy of a molecular system as a function of its structure (and forces via the gradient of the energy with respect to those atomic coordinates) is referred to as a potential energy surface (PES). In general, MD simulations integrate the equations of motion using a PES from one of two sources: (1) Classical MD using the force fields, which are parameterized equations that approximate the true PES, and are less costly to evaluate, allowing for the treatment of larger systems and longer timesteps. (2) *ab-inito* MD (which calculates the energy of a molecular system via electronic structure methods, *e.g.*, DFT) provide more accurate PES, but are limited in the system size and timesteps that are practically accessible due to the cost of evaluating the PES at a given point.

A.2 Related Works

SE(3)-Equivariant Representation for Small Molecules and Proteins. The molecular systems are indeed a set of atoms located in the 3D Euclidean space. From a machine learning point of view, the representation function or encoding function of such molecular systems needs to be group-symmetric, *i.e.*, the representation needs to be equivariant when we rotate or translate the whole system. Such symmetry is called the SE(3)-equivariance. Recently works [**zhang2023artificial**, 11] on molecules have provided a unified way of equivariant geometric modeling. They categorize the mainstream representation methods into three big venues: SE(3)-invariant models, SE(3)-equivariant models with spherical frame basis, and SE(3)-equivariant models with vector frame basis. (1) Invariant models that utilize invariant features (distances and angles) to predict the energies [12, 13], but the derived forces are challenging for ML optimization after integration. (2) Equivariant models with spherical frames that include a computationally expensive tensor-product operation [14, 15], which is unsuitable for large molecular systems. (3) Equivariant models with vector frames that have been explored for single stable molecules, including molecule representation and pretraining [16, 17, 18, 19], molecule conformation generation [20], protein representation [21], and protein folding and design [22, 23]. However, no one has tried it for binding complexes.

ML for Potential Energy and Force Learning for MD Simulation. One straightforward way of molecular dynamics (MD) simulation is through potential energy modeling. *Numerical methods* for MD simulation can be classified into classical MD and *ab-initio* MD, depending on using the classical mechanism or quantum mechanism to calculate the forces. Alternatively, a machine learning (ML) research line is to adopt geometric representation methods to learn the energies or the forces, *e.g.*, by the geometric methods listed above. The first work is DeePMD [1], which targets learning the potential energy function at each conformation. For inference, the learned energy can be applied to update the atom placement using i-PI software [24], composing the MD trajectories. TorchMD [25] utilizes TorchMD-Net [26] for energy prediction, which will be fed into the velocity Verlet algorithm for MD simulation. Similarly, Musaelian et al. adopts Allegro [28] model to learn the force at each conformation. The learned model will be used for MD trajectory simulation using LAMMPS [29]. In theory, all the geometric models on small molecules [16, 18, 19] and proteins [21] can be applied to the MD simulation task. However, there are two main challenges: (1) They require the interval between snapshots to be at the femtoseconds (1e-15 seconds) level for integration to effectively capture the motion of the molecules. (2) They take the position-energy pairs independently, and thus, they ignore their temporal correlations during learning.

We would like to kindly point out that the first version of our work was released in September 2023, and AI2BMD [30] is indeed a parallel work. Besides, we also checked if AI2BMD open-sourced the codes, and the authors confirmed on Jan 14th, 2025, that "The current version supports single-chain protein simulations, while more functions will be added and released later. Please check this link for more details. Meanwhile, we would also like to point out that the problem formulation of AI2BMD is **ML for Potential Energy and Force Learning for MD Simulation** while ours is in the **ML for Trajectory Learning for MD Simulation** research line.

ML for Trajectory Learning for MD Simulation. More recent works have explored MD simulation by directly learning the coordinates along the trajectories. There are two key differences between energy and trajectory prediction for MD: (1)

Supplementary Table S1. Comparison of different numerical and machine learning (ML) methods for molecular dynamics (MD). AR for autoregressive and denoising for the denoising diffusion method.

Category	Method	Energy / Force Calculation	Dynamics	Objective Function	Publications
Numerical Methods	Classical MD	Classical Mechanics: Force Field	Newtonian Dynamics	–	–
	Ab-initio MD	Quantum Mechanics: DFT for Schrodinger Equation	Newtonian Dynamics	–	–
	Langevin MD	Classical Mechanics: Force Field	Langevin Dynamics	–	–
ML Methods	DeePMD [1]	Atom-level Modeling	Newtonian Dynamics (i-PI)	Energy Prediction	PRL'18
	TorchMD [25]	Atom-level Modeling	Newtonian Dynamics (velocity Verlet)	Energy Prediction	ACS'20
	Allegro-LAMMPS [27]	Atom-level Modeling	Newtonian Dynamics (LAMMPS)	Force Prediction	ArXiv'23
	**VerletMD (Ours, baseline)**	Atom-level Modeling	Newtonian Dynamics (velocity Verlet)	Energy Prediction	–
	CGDMS [31]	Atom-level Modeling	Newtonian Dynamics (velocity Verlet)	Position Prediction	PLOS'21
	DiffMD [32]	Atom-level Modeling	AR + Denoising	Position Prediction	AAAI'23
	DFF [3]	Atom-level Modeling	AR + Denoising	Position Prediction	ACS'23
	CG-MD [6]	Atom-level Modeling	AR + Denoising	Position Prediction	TMLR'23
	**LigandMD (Ours, baseline)**	Atom-level Modeling	AR + Denoising	Position Prediction	–
	**NeuralMD ODE (Ours)**	Atom-level Modeling	Newtonian Dynamics	Position Prediction	–
	**NeuralMD SDE (Ours)**	Atom-level Modeling	Langevin Dynamics	Position Prediction	–

Energy prediction takes each conformation and energy as IID, while trajectory learning optimizes the conformations along the whole trajectory, enforcing the temporal relation. (2) The trajectory learning is agnostic to the magnitude of the timesteps, and energy prediction can be sensitive to longer-timestep MD simulations. More concretely, along such a trajectory learning research line, CGDMS [31] builds an SE(3)-invariant model, followed by the velocity Verlet algorithm for MD simulation. DiffMD [32] is a Markovian method and treats the dynamics between two consecutive snapshots as a coordinate denoising process. It then applies the SDE solver [33] to solve the molecular dynamics. A parallel work, DFF [3], applies a similar idea for MD simulation. CG-MD [6] encodes a hierarchical graph neural network model for an auto-regressive position generation and then adopts the denoising method for fine-tuning. However, these works disregard the prior knowledge of the Newtonian mechanics governing the motion of atoms.

MD Simulation in Protein-Ligand Binding. The MD simulation papers discussed so far are mainly for small molecules or proteins, not the binding dynamics. On the other hand, many works have studied the protein-ligand binding problem in the equilibrium state [34, 35, 36, 37], but not the dynamics. In this work, we consider a more challenging task, which is the protein-ligand binding dynamics. The viability of this work is also attributed to the efforts of the scientific community, where more binding dynamics datasets have been revealed, including PLAS-5k [9], MISATO [8], and PLAS-20k [38].

B Group Symmetry and Equivariance

In this article, a 3D molecular graph is represented by a collection of 3D point clouds. The corresponding symmetry group is SE(3), which consists of translations and rotations. Recall that we define the notion of equivariance functions in $\mathbf{R}^3$ in the main text through group actions. Formally, the group SE(3) is said to act on $\mathbf{R}^3$ if there is a mapping $\phi : \mathrm{SE}(3) \times \mathbf{R}^3 \to \mathbf{R}^3$ satisfying the following two conditions:

1. if $e \in \mathrm{SE}(3)$ is the identity element, then
$$\phi(e, r) = r \quad \text{for } \forall r \in \mathbf{R}^3.$$
2. if $g_1, g_2 \in \mathrm{SE}(3)$, then
$$\phi(g_1, \phi(g_2, r)) = \phi(g_1 g_2, r) \quad \text{for } \forall r \in \mathbf{R}^3.$$

Then, there is a natural SE(3) action on vectors r in $\mathbf{R}^3$ by translating r and rotating r for multiple times. For $g \in \mathrm{SE}(3)$ and $r \in \mathbf{R}^3$, we denote this action by gr. Once the notion of group action is defined, we say a function $f : \mathbf{R}^3 \to \mathbf{R}^3$ that transforms $r \in \mathbf{R}^3$ is equivariant if:
$$f(gr) = gf(r), \quad \text{for } \forall \ r \in \mathbf{R}^3.$$
On the other hand, $f : \mathbf{R}^3 \to \mathbf{R}^1$ is invariant, if f is independent of the group actions:
$$f(gr) = f(r), \quad \text{for } \forall \ r \in \mathbf{R}^3.$$

For some scenarios, our problem is chiral sensitive. That is, after mirror reflecting a 3D molecule, the properties of the molecule may change dramatically. In these cases, it's crucial to include reflection transformations into consideration. More precisely, we say an SE(3) equivariant function f is **reflection anti-symmetric**, if:
$$f(\rho r) \neq f(r), \tag{1}$$
for reflection $\rho \in \mathrm{E}(3)$.

C Equivariant Modeling With Vector Frames

Frame is a popular terminology in science areas. In physics, the frame is equivalent to a coordinate system. For example, we may assign a frame to all observers, although different observers may collect different data under different frames, the underlying physics law should be the same. In other words, denote the physics law by f, then f should be an equivariant function.

There are certain ways to choose the frame basis, and below we introduce two main types: the orthogonal basis and the protein backbone basis. The orthogonal basis can be built for flexible 3D point clouds such as atoms, while the protein backbone basis is specifically proposed to capture the protein backbone.

C.1 Basis

Since there are three orthogonal directions in $\mathbf{R}^3$, one natural frame in $\mathbf{R}^3$ can be a frame consisting of three orthogonal vectors:

$$F = (e_1, e_2, e_3).$$

Once equipped with a frame (coordinate system), we can project all geometric quantities to this frame. For example, an abstract vector $x \in \mathbf{R}^3$ can be written as $x = (r_1, r_2, r_3)$ under the frame F, if: $x = r_1 e_1 + r_2 e_2 + r_3 e_3$. A vector frame further requires the three orthonormal vectors in (e_1, e_2, e_3) to be equivariant. Intuitively, a vector frame will transform according to the global rotation or translation of the whole system. Once equipped with a vector frame, we can project vectors into this frame in an equivariant way:

$$x = \tilde{r}_1 e_1 + \tilde{r}_2 e_2 + \tilde{r}_3 e_3. \tag{2}$$

We call the process of $x \to \tilde{r} := (\tilde{r}_1, \tilde{r}_2, \tilde{r}_3)$ the **scalarization** or **projection** operation. Since $\tilde{r}_i = e_i \cdot x$ is expressed as an inner product between vector vectors, we know that $\tilde{r}$ consists of scalars.

In this article, we assign a vector frame to each node/edge, therefore we call them the local frames. We want to highlight that, in this section, we prove the equivariance property of the vector frame basis using the Gram-Schmidt project. However, the similar equivariance property can be easily guaranteed for the vector frame bases in the main article after we remove the mass center of the molecular system.

In the main body, we constructed three vector frames based on three granularities. Here we provide the proof on the protein backbone frame. Say the three backbone atoms in on proteins are x_i, x_j, x_k respectively. Then the vector frame is defined by:

$$\text{Vector-Frame}(x_i, x_j) := \textbf{Gram-Schmidt}\{x_i - x_j, x_i - x_k, (x_i - x_j) \times (x_i - x_k)\}. \tag{3}$$

The Gram-Schmidt orthogonalization makes sure that the Vector-Frame(x_i, x_j) is orthonormal.

Reflection Antisymmetric Since we implement the cross product $\times$ for building the local frames, the third vector in the frame is a pseudo-vector. Then, the **projection** operation is not invariant under reflections (the inner product between a vector and a pseudo-vector change signs under reflection). Therefore, our model can discriminate two 3D geometries with different chiralities.

Our local frames also enable us to output vectors equivariantly by multiplying scalars (v_1, v_2, v_3) with the frame: $v = v_1 \cdot e_1 + v_2 \cdot e_2 + v_3 \cdot e_3$.

Equivariance w.r.t. cross-product The goal is to prove that the cross-product is equivariant to the SE(3)-group, *i.e.*:

$$gx \times gy = g(x \times y), \qquad g \in \text{SE(3)-Group} \tag{4}$$

Proof. **Geometric proof.** From intuition, with rotation matrix g, we are transforming the whole basis, thus the direction of $gx \times gy$ changes equivalently with g. And for the value/length of $gx \times gy$, because $|gx \times gy| = \|gx\| \cdot \|gy\| \cdot \sin\theta = \|x\| \cdot \|y\| \cdot \sin\theta = |x \times y|$. So the length stays the same, and the direction changes equivalently. Intuitively, this interpretation is quite straightforward.

Analytical proof. A more rigorous proof can be found below:

First, we have that for the rotation matrix g:

$$gx \times gy = \begin{bmatrix} g_1^T x \\ g_2^T x \\ g_3^T x \end{bmatrix} \times \begin{bmatrix} g_1^T y \\ g_2^T y \\ g_3^T y \end{bmatrix} = \begin{bmatrix} g_2^T x \cdot g_3^T y - g_3^T x \cdot g_2^T y \\ -g_1^T x \cdot g_3^T y + g_3^T x \cdot g_1^T y \\ g_1^T x \cdot g_2^T y - g_2^T x \cdot g_1^T y \end{bmatrix}, \tag{5}$$

where $g_i, x, y \in \mathbb{R}^{3\times 1}$.

Because $A^T C \cdot B^T D - A^T D \cdot B^T C = (A \times B)^T (C \times D)$, so we can have:

$$gx \times gy = \begin{bmatrix} g_2^T x \cdot g_3^T y - g_3^T x \cdot g_2^T y \\ -g_1^T x \cdot g_3^T y + g_3^T x \cdot g_1^T y \\ g_1^T x \cdot g_2^T y - g_2^T x \cdot g_1^T y \end{bmatrix} = \begin{bmatrix} (g_2 \times g_3)^T (x \times y) \\ (g_3 \times g_1)^T (x \times y) \\ (g_1 \times g_2)^T (x \times y). \end{bmatrix} \tag{6}$$

Then because:

$$\begin{aligned} &\det(g) = (g_2 \times g_3)^T g_1 = g_1^T g_1 = 1 \\ \Longrightarrow &(g_2 \times g_3)^T g_1 g_1^{-1} = g_1^T g_1 g_1^{-1} \\ \Longrightarrow &(g_2 \times g_3)^T = g_1^T. \end{aligned} \tag{7}$$

Thus, we can have

$$gx \times gy = \begin{bmatrix} (g_2 \times g_3)^T (x \times y) \\ (g_3 \times g_1)^T (x \times y) \\ (g_1 \times g_2)^T (x \times y) \end{bmatrix} = \begin{bmatrix} g_1^T (x \times y) \\ g_2^T (x \times y) \\ g_3^T (x \times y) \end{bmatrix} = g(x \times y). \tag{8}$$

□

Rotation symmetric The goal is to prove

$$\text{Vector-Frame}(gx_i, gx_j) = g\textbf{Gram-Schmidt}\{x_i - x_j, x_i - x_k, (x_i - x_j) \times (x_i - x_k)\}. \tag{9}$$

Proof. We can have:

$$\begin{aligned} \text{Vector-Frame}(gx_i, gx_j) &= \textbf{Gram-Schmidt}\{gx_i - gx_j, gx_i - gx_k, (gx_i - gx_j) \times (gx_i - gx_k)\} \\ &= \textbf{Gram-Schmidt}\{g(x_i - x_j), g(x_i - x_k), g((x_i - x_j) \times (x_i - x_k))\}. \end{aligned} \tag{10}$$

Recall that Gram-Schmidt projection ($\textbf{Gram-Schmidt}\{v_1, v_2, v_3\}$) is:

$$\begin{aligned} u_1 &= v_1, & e_1 &= \frac{v_1}{\|v_1\|}, \\ u_2 &= v_2 - \frac{u_1^T v_2}{\|u_1\|} u_1, & e_2 &= \frac{v_2}{\|v_2\|}, \\ u_3 &= v_3 - \frac{u_1^T v_3}{\|u_1\|} u_1 - \frac{u_2^T v_3}{\|u_2\|} u_2, & e_3 &= \frac{v_3}{\|v_3\|}. \end{aligned} \tag{11}$$

Thus, the Gram-Schmidt projection on the rotated vector ($\textbf{Gram-Schmidt}\{gv_1, gv_2, gv_3\}$) is:

$$\begin{aligned} u_1' &= gv_1, \\ u_2' &= gv_2 - g\frac{u_1^T v_2}{\|u_1\|} u_1, \\ u_3' &= gv_3 - g\frac{u_1^T v_3}{\|u_1\|} u_1 - g\frac{u_2^T v_3}{\|u_2\|} u_2, \end{aligned} \tag{12}$$

Thus, $\textbf{Gram-Schmidt}\{gv_1, gv_2, gv_3\} = g\textbf{Gram-Schmidt}\{v_1, v_2, v_3\}$.

□

Transition symmetric

$$\text{Vector-Frame}(x_i + \delta x, x_j + \delta x) = \textbf{Gram-Schmidt}\{x_i - x_j, x_i - x_k, (x_i - x_j) \times (x_i - x_k)\}. \tag{13}$$

Proof. Because the basis is based on the difference of coordinates, it is straightforward to observe that **Gram-Schmidt**$\{v_1 + t, v_2 + t, v_3 + t\}$ = **Gram-Schmidt**$\{v_1, v_2, v_3\}$. So the frame operation is transition equivariant. We also want to highlight that for all the other vector frame bases introduced in the main article, we remove the mass center for each molecular system, thus, we can guarantee the transition equivariance property. □

Reflection antisymmetric

$$\text{Vector-Frame}(x_i, x_j) \neq \text{Vector-Frame}(-x_i, -x_j). \tag{14}$$

Proof. From intuition, this makes sense because the cross-product is anti-symmetric.

A simple counter-example is the original geometry R and the reflected geometry by the original point $-R$. Thus the two bases before and after the reflection group is the following:

$$\textbf{Gram-Schmidt}\{x_i - x_j, x_i - x_k, (x_i - x_j) \times (x_i - x_k)\} \tag{15}$$
$$\textbf{Gram-Schmidt}\{-x_i + x_j, -x_i + x_k, (x_i - x_j) \times (x_i - x_k)\}. \tag{16}$$

The bases between v_1, v_2, v_3 and $\{-v_1, -v_2, v_3\}\}$ are different, thus such frame construction is reflection anti-symmetric.
□

If you are able to get the above derivations, then you can tell that this can be trivially generalized to arbitrary vector frames as long as the three bases are non-coplanar.

C.2 Scalarization

Once we have the three vectors as the vector frame basis, the next step is modeling. Scalarization refers to the function in which we map the vectors to the frames or bases we construct. Suppose the frame is $\mathscr{F} = (e_1, e_2, e_3)$, then for a vector (tensor) h, the corresponding scalarization is:

$$h \odot \mathscr{F} = (h \odot e_1, h \odot e_2, h \odot e_3) = (h_1, h_2, h_3). \tag{17}$$

D MISATO Dataset Specifications

In this section, we provide more details on the MISATO dataset [8]. Note that for small molecule ligands, we ignore the Hydrogen atoms.

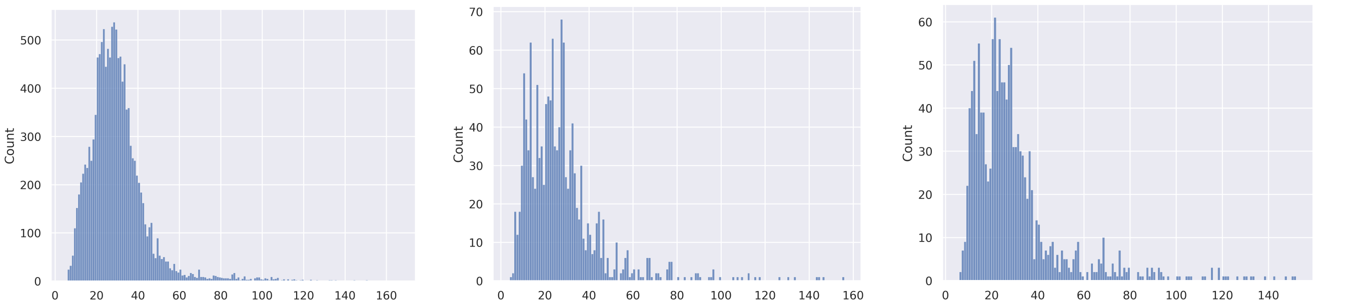

(a) Training data. **(b)** Validation data. **(c)** Test data.

Supplementary Figure S1. Distribution on # atoms in small molecule ligands for all protein-ligand complex. Source data are provided as a Source Data file.

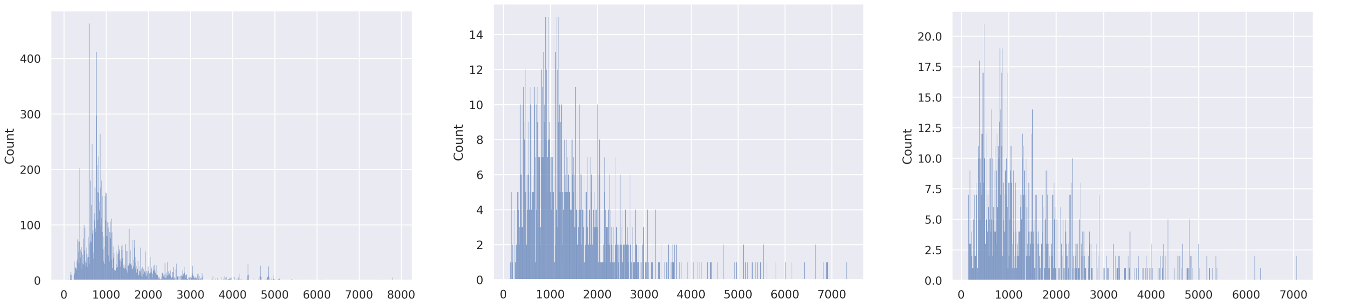

(a) Training data. **(b)** Validation data. **(c)** Test data.

Supplementary Figure S2. Distribution on # residues in proteins for all protein-ligand complex. Source data are provided as a Source Data file.

E Details of NeuralMD

E.1 Model Architecture and Hyperparameters

In this section, we provide more details on the model architecture in Figure S3, and hyperparameter details in Table S2.

First, we explain each of the three modules in detail and list the dimensions of each variable to make it easier for readers to understand. Suppose the representation dimension is d.

BindingNet-Ligand:

- $z^{(l)} = \text{Embedding}(f^{(l)}) \in \mathbb{R}^{N_{\text{atom}} \cdot d}$ is atom type embedding.
- Then for each atom type embedding $z^{(l)}$, we add a normalization by multiplying it with the RBF of distance among the neighborhoods, and the resulting atom type embedding stays the same dimension $z^{(l)} \in \mathbb{R}^{N_{\text{atom}} \times d}$.
- $\{h_i^{(l)} = \text{Agg}_j(x_i^{(l)} - x_j^{(l)}) \cdot z_i^{(l)}\} \in \mathbb{R}^{N_{\text{atom}} \cdot d \cdot 3}$ is the equivariant representation of each atom.
- $\{h_{ij}^{(l)}\} \in \mathbb{R}^{N_{\text{edge}} \cdot 2d \cdot 3}$ is the invariant representation after scalarization. Then we will take a simple sum-pooling, followed by an MLP to get the invariant representation $h_{ij}^{(l)} \in \mathbb{R}^{N_{\text{edge}} \cdot d}$.
- Finally, we will repeat L layers of MPNN:

$$
\begin{aligned}
\text{vec}_i^{(l)} &= \text{vec}_i^{(l)} + \text{Agg}_j\big(\text{vec}_i^{(l)} \cdot \text{MLP}(h_{ij}) + (x_i^{(l)} - x_j^{(p)}) \cdot \text{MLP}(h_{ij})\big), \quad //\{\text{vec}_i^{(l)}\} \in \mathbb{R}^{N_{\text{atom}} \cdot 3} \\
h_i^{(l)} &= h_i^{(l)} + \text{Agg}_j\big(\text{MLP}(h_{ij})\big). \quad //\{h_i^{(l)}\} \in \mathbb{R}^{N_{\text{atom}} \cdot d}
\end{aligned}
\tag{18}
$$

BindingNet-Protein:

- $z^{(p)} \in \mathbb{R}^{N_{\text{backbone-atom}} \cdot d}$ is the backbone-atom type representation by aggregating the neighbors without the cutoff c.
- $\tilde{z}^{(p)} \in \mathbb{R}^{N_{\text{backbone-atom}} \cdot d}$ is the backbone-atom type representation.
- $\{h_i^{(p)}\} \in \mathbb{R}^{N_{\text{backbone-atom}} \cdot d \cdot 3}$ is the backbone-atom equivariant representation.
- $\{h_{ij}^{(p)}\} \in \mathbb{R}^{N_{\text{edge}} \cdot 2d \cdot 3}$ is the invariant representation after scalarization. Then we take a simple sum-pooling, followed by an MLP to get the invariant representation $\{h_{ij}^{(l)}\} \in \mathbb{R}^{N_{\text{edge}} \cdot d}$.
- Finally, we get the residue-level representation as $h^{(p)} = \tilde{z}^{(p)} + (h_{N,C\alpha}^{(p)} + h_{C\alpha,C}^{(p)})/2 \in \mathbb{R}^{N_{\text{residue}} \cdot d}$.

BindingNet-Complex:

- $\{h_{ij}\} \in \mathbb{R}^{N_{\text{edge}} \cdot d \cdot 3}$ is the equivariant interaction/edge representation.
- $\{h_{ij} = h_{ij} \cdot \mathscr{F}_{\text{complex}}\} \mathbb{R}^{N_{\text{edge}} \cdot d \cdot 3}$ is the scalarization. Then we take a simple sum-pooling, followed by an MLP to get the invariant representation $\{h_{ij}^{(l)}\} \in \mathbb{R}^{N_{\text{edge}} \cdot d}$.
- The final output is obtained by L MPNN layers as:

$$
\begin{aligned}
\text{vec}_{ij}^{(pl)} &= \text{vec}_i^{(l)} \cdot \text{MLP}(h_{ij}) + (x_i^{(l)} - x_j^{(p)}) \cdot \text{MLP}(h_{ij}), \quad //\{\text{vec}_{ij}^{(pl)}\} \in \mathbb{R}^{N_{\text{edge}} \cdot 3} \\
F_i^{(l)} &= \text{vec}_i^{(l)} + \text{Agg}_{j \in \mathcal{N}(i)} \text{vec}_{ij}^{(pl)}. \quad //\{F_i^{(l)}\} \in \mathbb{R}^{N_{\text{atom}} \cdot 3}
\end{aligned}
\tag{19}
$$

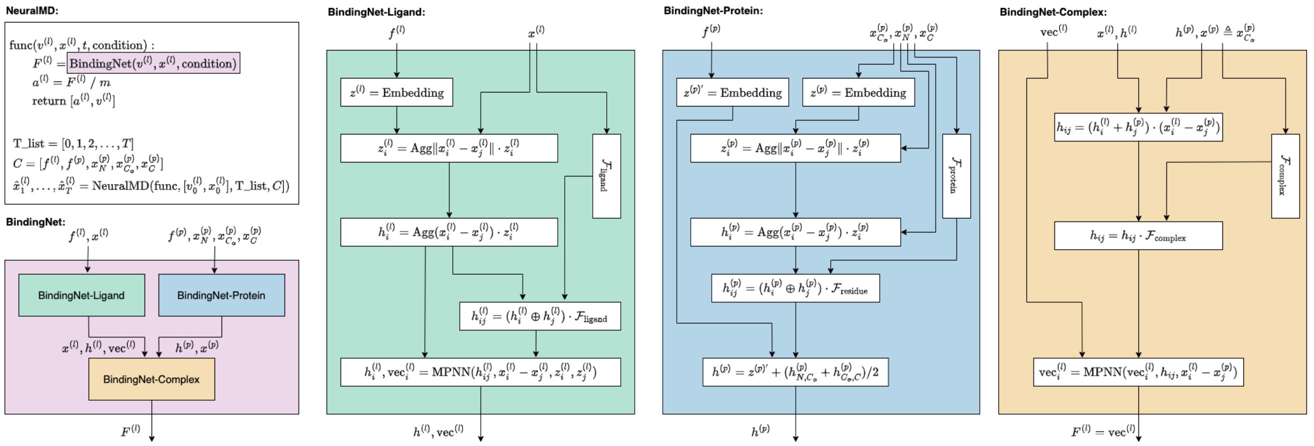

Supplementary Figure S3. Detailed pipeline of NeuralMD ODE. In the three key modules of BindingNet, there are three vertical boxes, corresponding to three granularities of vector frames.

Supplementary Table S2. Hyperparameter specifications for NeuralMD.

Hyperparameter	Value
# layers	{5}
cutoff c	{5}
velocity initial mapping function	{True, False}
velocity refinement coefficient α	{0, 0.01, 0.001}
step size (integration)	{0.25, 0.5, 1}
learning rate	{1e-3, 1e-4}
optimizer	{SGD, Adam }

E.2 Overdamped, Underdamped, and Data-driven Langevin Dynamics

In the literature on using Langevin dynamics for MD simulation, there are two main categories: **overdamped Langevin dynamics** and **underdamped Langevin dynamics**. In this section, we discuss these methods and their applicable settings.

Langevin dynamics or damped Langevin dynamics is defined as

$$ma = -\nabla U(x) - \gamma m v + \sqrt{2m\gamma k_B T} R(t), \tag{20}$$

where γ is the damping constant or collision frequency, T is the temperature, k_B is the Boltzmann's constant, and $R(t)$ is a delta-correlated stationary Gaussian process with zero-mean.

Overdamped Langevin dynamics (Brownian dynamics) This is the friction-dominated regime, where the inertia term (ma) is negligible compared to the friction term ($-\gamma m v$). The equation for overdamped Langevin dynamics is:

$$-\nabla U(x) - \gamma m v + \sqrt{2m\gamma k_B T} R(t) = 0. \tag{21}$$

Thus, the trajectories are given by:

$$\begin{aligned} x_{t+1} - x_t &= -\frac{1}{\gamma m}\nabla U(x) + \frac{\sqrt{2m\gamma k_B T}}{\gamma m} R(t) \\ &= -\frac{D}{k_B T}\nabla U(X) + \sqrt{2D} R(t), \end{aligned} \tag{22}$$

where $D = k_B T/\gamma$. This has been widely used in deep generative models like Denoising Diffusion Probabilistic Model (DDPM) [39]. Specifically, it is employed as the Monte Carlo sampling step.

For application, this is suitable for large molecular systems like protein folding in solution because the motion is slow and dominated by viscous drag. However, such a dynamic is not suitable for simulating small particles like small molecules.

Underdamped Langevin dynamics This is the inertia-dominated regime, where the inertia term (ma) is comparable to or larger than the friction term ($-\gamma m v$). It is applicable for describing a particle moving in a low-viscosity medium or when the friction coefficient γ is small, allowing the particle to exhibit significant inertial motion.

For application, underdamped Langevin dynamics is ideal for systems where inertia plays an important role, such as in small molecules or systems with oscillations or high-frequency dynamics. This regime is typically used in molecular dynamics (MD) simulations where inertia is significant, such as simulating vibrational modes of molecules or dynamics in a gas phase.

Data-driven Langevin dynamics in NeuralMD The Langevin dynamics modeled in NeuralMD make no prior assumptions about overdamped or underdamped behavior. Instead, we learn a data-driven approximation of Equation (20):

$$ma = \text{BindingNet}(f^{(l)}, x^{(l)}, f^{(p)}, x_N^{(p)}, x_{C_\alpha}^{(p)}, x_C^{(p)}) + \text{BindingNet-Ligand}(f^{(l)}, x^{(l)}) \cdot \varepsilon, \tag{23}$$

where we are using the reparameterization trick, and ε is sampled from a standard Gaussian.

Summary To summarize, if we treat the MD simulation of small particles as a density estimation task, there are several solutions available, including DDPM-like DenoisingLD and NeuralMD. However, if we consider these models as learning or simulating dynamics from a physics perspective, we must be cautious. Current DDPM methods like DenoisingLD rely on overdamped Langevin dynamics, which are not well-suited for simulating small molecule dynamics. In this context, NeuralMD is a more accurate option.

E.3 Simultaneous Integration

The goal is to calculate the second-order integration, as Equation 12 in the manuscript:

$$a_\tau^{(l)} = \frac{F_\tau^{(l)}}{m}, \qquad \hat{v}_t^{(l)} = v_0^{(l)} + \int_0^t a_\tau^{(l)} d\tau, \qquad \hat{x}_t^{(l)} = x_0^{(l)} + \int_0^t \hat{v}_\tau^{(l)} d\tau. \tag{24}$$

Case 1: non-simultaneous integration. In this case, we need to call the ODE/SDE function twice. The first ODE/SDE function call corresponds to solving the acceleration integration with $\big[dv/dt\big] = \big[F/m\big]$. This means that if we are integrating for T timesteps, then we have

$$\begin{aligned} v_1 &= \text{ODE/SDE Integration}(\text{model}(x_0)/m), \\ v_2 &= \text{ODE/SDE Integration}(\text{model}(x_1)/m), \\ &\ldots \\ v_T &= \text{ODE/SDE Integration}(\text{model}(x_{T-1})/m). \end{aligned} \tag{25}$$

The second ODE/SDE call function call corresponding to solving the velocity integration with $\big[dx/dt\big] = \big[v\big]$. Similarly, this means that if we are integrating for T timesteps, then we have:

$$\begin{aligned} x_1 &= \text{ODE/SDE Integration}(v_0), \\ x_2 &= \text{ODE/SDE Integration}(v_1), \\ &\ldots \\ x_T &= \text{ODE/SDE Integration}(v_{T-1}). \end{aligned} \tag{26}$$

Thus, we will call the ODE/SDE integration calls **twice**, as in Equations (25) and (26).

Case 2: simultaneous integration. The case means that we can call the acceleration integration and velocity integration simultaneously, as shown in Equation 14 of the manuscript, we utilize

$$\begin{bmatrix} dx/dt \\ dv/dt \end{bmatrix} = \begin{bmatrix} v \\ F/m \end{bmatrix}. \tag{27}$$

Then we call the integration for T timesteps as:

$$\begin{aligned} \begin{bmatrix} v_1 \\ x_1 \end{bmatrix} &= \text{ODE/SDE Integration}(\begin{bmatrix} \text{model}(x_0)/m \\ v_0 \end{bmatrix}), \\ \begin{bmatrix} v_2 \\ x_2 \end{bmatrix} &= \text{ODE/SDE Integration}(\begin{bmatrix} \text{model}(x_1)/m \\ v_1 \end{bmatrix}), \\ &\ldots \\ \begin{bmatrix} v_T \\ x_T \end{bmatrix} &= \text{ODE/SDE Integration}(\begin{bmatrix} \text{model}(x_{T-1})/m \\ v_{T-1} \end{bmatrix}). \end{aligned} \tag{28}$$

Thus, we will call the ODE/SDE integration calls just **once**, in comparison to twice in the first case (Equations (25) and (26)), resulting in a twofold increase in efficiency.

F Empirical Results

F.1 Standard Deviation for Multi-trajectory Semi-flexible Binding Prediction

We are running all the experiments using three random seeds, 0, 42, and 123. In the tables of the main article, we are only reporting the mean due to the space limitation. Thus here, we would also like to report the results with standard deviation. We show the reconstruction and validity results on three multi-trajectory binding dynamics: MISATO-100 in Table S3, MISATO-1000 in Table S4, and MISATO-All in Table S5.

Supplementary Table S3. Results of multi-trajectory binding dynamics predictions on MISATO-100. Four evaluation metrics are considered: MAE (Å, ↓), RMSE (↓), Matching(↓), and Stability (%, ↑). Source data are provided as a Source Data file.

	Reconstruction		Validity	
	MAE	RMSE	Matching	Stability
VerletMD	85.286 ± 0.035	54.996 ± 0.17	46.753 ± 2.05	10.051 ± 1.39
GNN-MD	5.964 ± 0.05	3.938 ± 0.02	0.671 ± 0.02	70.546 ± 1.81
DenoisingLD	8.251 ± 0.02	5.541 ± 0.01	1.744 ± 0.04	29.545 ± 0.68
NeuralMD ODE (ours)	5.867 ± 0.00	3.870 ± 0.00	0.539 ± 0.02	79.553 ± 2.17
NeuralMD SDE (ours)	5.868 ± 0.00	3.871 ± 0.00	0.533 ± 0.02	80.229 ± 1.23

Supplementary Table S4. Results of multi-trajectory binding dynamics predictions on MISATO-1000. Four evaluation metrics are considered: MAE (Å, ↓), RMSE (↓), Matching(↓), and Stability (%, ↑). Source data are provided as a Source Data file.

	Reconstruction		Validity	
	MAE	RMSE	Matching	Stability
VerletMD	104.537 ± 0.07	68.942 ± 0.04	48.899 ± 0.19	10.574 ± 0.59
GNN-MD	7.524 ± 0.01	4.915 ± 0.01	0.670 ± 0.05	68.310 ± 4.08
DenoisingLD	9.251 ± 0.15	6.074 ± 0.10	1.362 ± 0.06	37.289 ± 1.54
NeuralMD ODE (ours)	7.459 ± 0.00	4.867 ± 0.00	0.612 ± 0.02	70.362 ± 1.99
NeuralMD SDE (ours)	7.476 ± 0.00	4.876 ± 0.00	0.457 ± 0.00	83.960 ± 0.00

Supplementary Table S5. Results of multi-trajectory binding dynamics predictions on MISATO-All. Four evaluation metrics are considered: MAE (Å, ↓), RMSE (↓), Matching(↓), and Stability (%, ↑). Source data are provided as a Source Data file.

	Reconstruction		Validity	
	MAE	RMSE	Matching	Stability
VerletMD	97.213 ± 0.29	64.405 ± 0.19	50.857 ± 0.67	11.888 ± 1.49
GNN-MD	7.637 ± 0.01	5.048 ± 0.00	0.675 ± 0.02	69.244 ± 1.65
DenoisingLD	8.149 ± 0.79	5.387 ± 0.52	0.764 ± 0.38	68.315 ± 20.01
NeuralMD ODE (ours)	7.513 ± 0.01	4.961 ± 0.00	0.491 ± 0.02	81.991 ± 1.80
NeuralMD SDE (ours)	7.517 ± 0.00	4.963 ± 0.00	0.474 ± 0.00	83.264 ± 0.00

F.2 Standard Deviation for Single-trajectory Semi-flexible Binding Prediction

We illustrate the reconstruction and validity results with standard deviation on ten single-trajectory binding dynamics in Table S6.

Supplementary Table S6. Results on ten single-trajectory binding dynamics prediction. Results with optimal training loss are reported. Four evaluation metrics are considered: MAE (Å, ↓), RMSE (↓), Matching (↓), and Stability (%, ↓). Source data are provided as a Source Data file.

		VerletMD	GNN MD	Denoising LD	NeuralMD (ODE)	NeuralMD (SDE)
5WIJ	MAE	14.629 ± 0.04	2.280 ± 0.05	2.501 ± 0.01	2.252 ± 0.16	2.260 ± 0.15
	RMSE	10.221 ± 0.03	1.521 ± 0.03	1.644 ± 0.00	1.514 ± 0.13	1.514 ± 0.10
	Matching	5.459 ± 0.10	0.803 ± 0.09	0.815 ± 0.07	0.464 ± 0.09	0.615 ± 0.18
	Stability	24.360 ± 0.52	54.475 ± 2.45	52.418 ± 4.65	82.046 ± 6.26	67.464 ± 14.15
4ZX0	MAE	21.278 ± 0.02	2.370 ± 0.09	3.138 ± 0.06	1.878 ± 0.00	2.158 ± 0.17
	RMSE	14.357 ± 0.01	1.599 ± 0.05	2.045 ± 0.03	1.263 ± 0.00	1.455 ± 0.14
	Matching	7.971 ± 0.05	0.555 ± 0.02	1.072 ± 0.03	0.428 ± 0.00	0.696 ± 0.09
	Stability	19.168 ± 0.35	68.613 ± 1.14	44.228 ± 0.78	81.401 ± 0.78	59.109 ± 7.87
3EOV	MAE	27.960 ± 0.06	3.512 ± 0.02	4.055 ± 0.01	3.858 ± 0.31	3.395 ± 0.22
	RMSE	18.821 ± 0.04	2.413 ± 0.02	2.787 ± 0.01	2.651 ± 0.20	2.309 ± 0.11
	Matching	13.588 ± 0.12	1.216 ± 0.05	1.209 ± 0.01	1.062 ± 0.06	0.962 ± 0.03
	Stability	13.067 ± 0.29	40.984 ± 2.58	41.469 ± 0.81	47.328 ± 4.95	50.108 ± 3.48
4K6W	MAE	15.428 ± 0.01	3.695 ± 0.04	3.942 ± 0.01	3.656 ± 0.12	3.765 ± 0.20
	RMSE	10.357 ± 0.01	2.402 ± 0.03	2.635 ± 0.00	2.400 ± 0.08	2.501 ± 0.17
	Matching	7.505 ± 0.06	1.038 ± 0.07	0.839 ± 0.01	0.928 ± 0.19	1.076 ± 0.49
	Stability	15.441 ± 0.13	42.480 ± 2.74	53.820 ± 0.57	49.438 ± 8.66	49.700 ± 12.15
1KTI	MAE	18.157 ± 0.04	6.641 ± 0.05	7.051 ± 0.02	6.675 ± 0.04	6.646 ± 0.01
	RMSE	12.723 ± 0.03	4.173 ± 0.04	4.369 ± 0.01	4.176 ± 0.05	4.141 ± 0.01
	Matching	7.467 ± 0.03	0.386 ± 0.03	0.268 ± 0.02	0.337 ± 0.17	0.167 ± 0.02
	Stability	19.352 ± 0.61	81.831 ± 2.03	91.986 ± 2.36	86.430 ± 11.59	98.508 ± 0.57
1XP6	MAE	13.753 ± 0.01	2.378 ± 0.06	2.218 ± 0.03	1.924 ± 0.05	2.061 ± 0.06
	RMSE	9.587 ± 0.01	1.561 ± 0.03	1.472 ± 0.02	1.280 ± 0.04	1.356 ± 0.05
	Matching	4.672 ± 0.08	0.966 ± 0.08	0.676 ± 0.01	0.537 ± 0.04	0.615 ± 0.11
	Stability	28.129 ± 1.17	49.239 ± 2.78	64.951 ± 0.33	75.533 ± 3.82	69.423 ± 6.48
4YUR	MAE	16.764 ± 0.03	7.031 ± 0.07	7.128 ± 0.01	6.957 ± 0.07	7.038 ± 0.19
	RMSE	11.069 ± 0.00	4.641 ± 0.07	4.807 ± 0.00	4.597 ± 0.03	4.679 ± 0.11
	Matching	9.555 ± 0.04	0.920 ± 0.02	0.834 ± 0.01	0.584 ± 0.07	0.749 ± 0.27
	Stability	16.542 ± 0.42	47.555 ± 1.06	49.676 ± 0.64	69.775 ± 6.88	60.344 ± 16.47
4G3E	MAE	5.111 ± 0.02	2.709 ± 0.08	3.588 ± 0.11	2.191 ± 0.02	2.345 ± 0.26
	RMSE	3.503 ± 0.01	1.785 ± 0.02	2.321 ± 0.07	1.453 ± 0.01	1.536 ± 0.16
	Matching	3.388 ± 0.03	0.893 ± 0.59	1.069 ± 0.09	0.505 ± 0.12	0.521 ± 0.03
	Stability	31.852 ± 0.79	61.802 ± 18.90	40.823 ± 4.19	71.436 ± 11.43	68.729 ± 2.78
6B7F	MAE	31.934 ± 0.01	4.136 ± 0.10	4.431 ± 0.01	3.921 ± 0.14	3.842 ± 0.06
	RMSE	22.168 ± 0.01	2.768 ± 0.08	3.047 ± 0.01	2.652 ± 0.11	2.601 ± 0.03
	Matching	21.691 ± 0.02	1.194 ± 0.13	0.672 ± 0.03	0.459 ± 0.14	0.741 ± 0.26
	Stability	11.050 ± 0.04	39.067 ± 5.15	61.583 ± 3.10	75.692 ± 11.97	57.917 ± 13.31
3B9S	MAE	19.473 ± 0.04	2.578 ± 0.08	2.811 ± 0.05	3.039 ± 0.52	3.132 ± 0.92
	RMSE	11.696 ± 0.03	1.699 ± 0.06	1.868 ± 0.04	1.999 ± 0.35	2.078 ± 0.62
	Matching	0.923 ± 0.15	1.414 ± 0.27	0.472 ± 0.03	0.659 ± 0.43	0.444 ± 0.15
	Stability	57.801 ± 3.84	49.306 ± 11.48	71.852 ± 1.40	76.065 ± 17.78	77.801 ± 11.54

Supplementary References

[1] Linfeng Zhang, Jiequn Han, Han Wang, Roberto Car, and EJPRL Weinan. "Deep potential molecular dynamics: a scalable model with the accuracy of quantum mechanics". In: *Physical Review Letters* 120.14 (2018), p. 143001.

[2] Dennis C Rapaport. *The art of molecular dynamics simulation*. Cambridge University Press, 2004.

[3] Marloes Arts, Victor Garcia Satorras, Chin-Wei Huang, Daniel Zugner, Marco Federici, Cecilia Clementi, Frank Noe, Robert Pinsler, and Rianne van den Berg. "Two for one: Diffusion models and force fields for coarse-grained molecular dynamics". In: *Journal of Chemical Theory and Computation* 19.18 (2023), pp. 6151–6159.

[4] Martin Karplus and John Kuriyan. "Molecular dynamics and protein function". In: *Proceedings of the National Academy of Sciences* 102.19 (2005), pp. 6679–6685.

[5] Hans Frauenfelder, Guo Chen, Joel Berendzen, Paul W Fenimore, Helén Jansson, Benjamin H McMahon, Izabela R Stroe, Jan Swenson, and Robert D Young. "A unified model of protein dynamics". In: *Proceedings of the National Academy of Sciences* 106.13 (2009), pp. 5129–5134.

[6] Xiang Fu, Tian Xie, Nathan J Rebello, Bradley D Olsen, and Tommi Jaakkola. "Simulate time-integrated coarse-grained molecular dynamics with geometric machine learning". In: *ICLR Workshop on Deep Generative Models for Highly Structured Data*. 2022.

[7] Shengchao Liu, Divin Yan, Hongyu Guo, and Anima Anandkumar. "An Equivariant Flow Matching Framework for Learning Molecular Crystallization". In: *ICML 2024 Workshop on Geometry-grounded Representation Learning and Generative Modeling*. 2024.

[8] Till Siebenmorgen, Filipe Menezes, Sabrina Benassou, Erinc Merdivan, Kieran Didi, André Santos Dias Mourão, Radosław Kitel, Pietro Liò, Stefan Kesselheim, Marie Piraud, et al. "MISATO: machine learning dataset of protein–ligand complexes for structure-based drug discovery". In: *Nature Computational Science* (2024), pp. 1–12.

[9] Divya B Korlepara, CS Vasavi, Shruti Jeurkar, Pradeep Kumar Pal, Subhajit Roy, Sarvesh Mehta, Shubham Sharma, Vishal Kumar, Charuvaka Muvva, Bhuvanesh Sridharan, et al. "Plas-5k: Dataset of protein-ligand affinities from molecular dynamics for machine learning applications". In: *Scientific Data* 9.1 (2022), p. 548.

[10] Daan Frenkel and Berend Smit. *Understanding molecular simulation: from algorithms to applications*. Academic Press San Diego, 2002.

[11] Shengchao Liu, Weitao Du, Yanjing Li, Zhuoxinran Li, Zhiling Zheng, Chenru Duan, Zhiming Ma, Omar Yaghi, Anima Anandkumar, Christian Borgs, Jennifer Chayes, Hongyu Guo, and Jian Tang. "Symmetry-Informed Geometric Representation for Molecules, Proteins, and Crystalline Materials". In: *Advances in Neural Information Processing Systems* 36 (2023), pp. 66084–66101.

[12] Kristof T Schütt, Huziel E Sauceda, P-J Kindermans, Alexandre Tkatchenko, and K-R Müller. "Schnet–a deep learning architecture for molecules and materials". In: *The Journal of Chemical Physics* 148.24 (2018), p. 241722.

[13] Johannes Klicpera, Shankari Giri, Johannes T Margraf, and Stephan Günnemann. "Fast and uncertainty-aware directional message passing for non-equilibrium molecules". In: *arXiv preprint arXiv:2011.14115* (2020).

[14] Tess Smidt, Nathaniel Thomas, Steven Kearnes, Lusann Yang, Li Li, Kai Kohlhoff, and Patrick Riley. "Tensor Field Networks: Rotation-and translation-equivariant neural networks for 3d point clouds". In: *arXiv preprint arXiv:1802.08219* (2018).

[15] Albert Musaelian, Simon Batzner, Anders Johansson, Lixin Sun, Cameron J Owen, Mordechai Kornbluth, and Boris Kozinsky. "Learning Local Equivariant Representations for Large-Scale Atomistic Dynamics". In: *Nature Communications* 14.1 (2023), p. 579.

[16] Shengchao Liu, Weitao Du, Zhi-Ming Ma, Hongyu Guo, and Jian Tang. "A group symmetric stochastic differential equation model for molecule multi-modal pretraining". In: *International Conference on Machine Learning*. PMLR. 2023, pp. 21497–21526.

[17] Weitao Du, Jiujiu Chen, Xuecang Zhang, Zhi-Ming Ma, and Shengchao Liu. "Molecule Joint Auto-Encoding: Trajectory Pretraining with 2D and 3D Diffusion". In: *Advances in Neural Information Processing Systems* 36 (2023), pp. 55077–55096.

[18] Victor Garcia Satorras, Emiel Hoogeboom, and Max Welling. "E(n) equivariant graph neural networks". In: (2021), pp. 9323–9332.

[19] Kristof T Schütt, Oliver T Unke, and Michael Gastegger. "Equivariant message passing for the prediction of tensorial properties and molecular spectra". In: (2021), pp. 9377–9388.

[20] Weitao Du, He Zhang, Yuanqi Du, Qi Meng, Wei Chen, Nanning Zheng, Bin Shao, and Tie-Yan Liu. "SE (3) equivariant graph neural networks with complete local frames". In: *International Conference on Machine Learning*. PMLR. 2022, pp. 5583–5608.

[21] Hehe Fan, Zhangyang Wang, Yi Yang, and Mohan Kankanhalli. "Continuous-Discrete Convolution for Geometry-Sequence Modeling in Proteins". In: *The Eleventh International Conference on Learning Representations*. 2022.

[22] John Ingraham, Vikas Garg, Regina Barzilay, and Tommi Jaakkola. "Generative models for graph-based protein design". In: *Advances in Neural Information Processing Systems* 32 (2019).

[23] John Jumper, Richard Evans, Alexander Pritzel, Tim Green, Michael Figurnov, Olaf Ronneberger, Kathryn Tunyasuvunakool, Russ Bates, Augustin Žídek, Anna Potapenko, et al. "Highly accurate protein structure prediction with AlphaFold". In: *Nature* 596.7873 (2021), pp. 583–589.

[24] Michele Ceriotti, Joshua More, and David E Manolopoulos. "i-PI: A Python interface for ab initio path integral molecular dynamics simulations". In: *Computer Physics Communications* 185.3 (2014), pp. 1019–1026.

[25] Stefan Doerr, Maciej Majewsk, Adrià Pérez, Andreas Krämer, Cecilia Clementi, Frank Noe, Toni Giorgino, and Gianni De Fabritiis. "TorchMD: A deep learning framework for molecular simulations". In: *Journal of Chemical Theory and Computation* 17.4 (2021), pp. 2355–2363.

[26] Philipp Tholke and Gianni De Fabritiis. "Equivariant Transformers for Neural Network based Molecular Potentials". In: *International Conference on Learning Representations*. 2022.

[27] Albert Musaelian, Anders Johansson, Simon Batzner, and Boris Kozinsky. "Scaling the leading accuracy of deep equivariant models to biomolecular simulations of realistic size". In: *Proceedings of the International Conference for High Performance Computing, Networking, Storage and Analysis*. 2023, pp. 1–12.

[28] Albert Musaelian, Simon Batzner, Anders Johansson, Lixin Sun, Cameron J Owen, Mordechai Kornbluth, and Boris Kozinsky. "Learning local equivariant representations for large-scale atomistic dynamics". In: *Nature Communications* 14.1 (2023), p. 579.

[29] Aidan P Thompson, H Metin Aktulga, Richard Berger, Dan S Bolintineanu, W Michael Brown, Paul S Crozier, Pieter J in't Veld, Axel Kohlmeyer, Stan G Moore, Trung Dac Nguyen, et al. "LAMMPS-a flexible simulation tool for particle-based materials modeling at the atomic, meso, and continuum scales". In: *Computer Physics Communications* 271 (2022), p. 108171.

[30] Tong Wang, Xinheng He, Mingyu Li, Yatao Li, Ran Bi, Yusong Wang, Chaoran Cheng, Xiangzhen Shen, Jiawei Meng, He Zhang, et al. "Ab initio characterization of protein molecular dynamics with AI2BMD". In: *Nature* (2024), pp. 1–9.

[31] Joe G Greener and David T Jones. "Differentiable molecular simulation can learn all the parameters in a coarse-grained force field for proteins". In: *PloS one* 16.9 (2021), e0256990.

[32] Fang Wu and Stan Z Li. "DIFFMD: A Geometric Diffusion Model for Molecular Dynamics Simulations". In: *Proceedings of the AAAI Conference on Artificial Intelligence*. Vol. 37. 4. 2023, pp. 5321–5329.

[33] Yang Song, Jascha Sohl-Dickstein, Diederik P Kingma, Abhishek Kumar, Stefano Ermon, and Ben Poole. "Score-Based Generative Modeling through Stochastic Differential Equations". In: *International Conference on Learning Representations*. 2021.

[34] Marta M Stepniewska-Dziubinska, Piotr Zielenkiewicz, and Pawel Siedlecki. "Development and evaluation of a deep learning model for protein–ligand binding affinity prediction". In: *Bioinformatics* 34.21 (2018), pp. 3666–3674.

[35] José Jiménez, Miha Skalic, Gerard Martinez-Rosell, and Gianni De Fabritiis. "K deep: protein–ligand absolute binding affinity prediction via 3d-convolutional neural networks". In: *Journal of Chemical Information and Modeling* 58.2 (2018), pp. 287–296.

[36] Derek Jones, Hyojin Kim, Xiaohua Zhang, Adam Zemla, Garrett Stevenson, WF Drew Bennett, Daniel Kirshner, Sergio E Wong, Felice C Lightstone, and Jonathan E Allen. "Improved protein–ligand binding affinity prediction with structure-based deep fusion inference". In: *Journal of chemical information and modeling* 61.4 (2021), pp. 1583–1592.

[37] Ziduo Yang, Weihe Zhong, Qiujie Lv, Tiejun Dong, and Calvin Yu-Chian Chen. "Geometric Interaction Graph Neural Network for Predicting Protein–Ligand Binding Affinities from 3D Structures (GIGN)". In: *The Journal of Physical Chemistry Letters* 14.8 (2023), pp. 2020–2033.

[38] U Deva Priyakumar, Divya B Korlepara, CS Vasavi, Rakesh Srivastava, Pradeep Kumar Pal, Saalim H Raza, Vishal Kumar, Shivam Pandit, Aathira G Nair, Sanjana Pandey, et al. "PLAS-20k: Extended Dataset of Protein-Ligand Affinities from MD Simulations for Machine Learning Applications". In: *Scientific Data* 11.1 (2024), p. 180.

[39] Jonathan Ho, Ajay Jain, and Pieter Abbeel. “Denoising diffusion probabilistic models”. In: *Advances in Neural Information Processing Systems* 33 (2020), pp. 6840–6851.
