# Supplementary material for: A multi-grained symmetric differential equation model for learning protein-ligand binding dynamics
Source: Nat Commun. 2025 Dec 30;17:1049. doi: 10.1038/s41467-025-67808-z (PMC12848303; doi:10.1038/s41467-025-67808-z)
Supplement: Supplementary file 2 — Description of Additonal Supplementary Files [file 41467_2025_67808_MOESM2_ESM.pdf]

Description of Additional Supplementary Files

File Name: Supplementary Video 1

Description: Demonstration of MD simulation.
